# Correlation between serum vitamin D level and acute invasive enteritis in children

**DOI:** 10.1002/iid3.70024

**Published:** 2024-09-24

**Authors:** Lingrong Yang, Yu Fang, Jinyu Zheng, Qiaoying Zhu, Li Tang, Fu Xiong

**Affiliations:** ^1^ Sichuan Provincial Maternity and Child Health Care Hospital Women and Children's Hospital of Chengdu Medical College Chengdu Sichuan China; ^2^ The Second School of Medicine Wenzhou Medical University Wenzhou Zhejiang China

**Keywords:** 25‐hydroxyvitamin D, acute invasive enteritis, children, infectious diarrhea, vitamin D

## Abstract

**Background:**

Diarrhea is a leading cause of death in young children worldwide. Vitamin D deficiency impairs the body's ability to clear pathogens, reduces tight junction protein expression in intestinal epithelial cells, and enhances Th1‐mediated intestinal inflammation. This study aimed to investigate the effects of serum vitamin D levels on acute invasive enteritis in children.

**Methods:**

This prospective cohort study included 82 children aged 1–3 years with clinically diagnosed acute invasive enteritis at Sichuan Maternal and Child Health Hospital from February 2021 to February 2022, alongside a control group of 80 healthy children. Fecal specimens were collected for routine tests and occult blood analysis, while blood samples were taken for routine tests, C‐reactive protein, and 25‐OHD levels. Comparative analyses were performed between groups, and multifactorial logistic regression was used to identify factors influencing invasive enteritis.

**Results:**

The study group showed significantly lower serum 25‐OHD levels (27.95 ± 9.91 ng/mL) compared to controls (32.76 ± 10.23 ng/mL, *p* < .01). Among the study group, 19.5% (16/82) had levels <20 ng/mL, versus 12.5% (10/80) in controls. Regular vitamin D supplementation was lower in the study group (58.5% vs. 77.5%, *p* < .05). Outdoor activity duration was also reduced (2.57 ± 0.98 h vs. 3.04 ± 0.88 h, *p* < .01). Multivariate analysis identified that exclusive breastfeeding, greater outdoor activity time and regular vitamin D supplementation were all associated with reduced risk of invasive enteritis (*p* < .05).

**Conclusion:**

The findings indicate an association between low serum 25‐OHD levels and acute invasive enteritis in children aged 1‐3 years, suggesting that consistent vitamin D supplementation and sufficient outdoor activity may protect against this condition.

## Graphical abstract

Low serum 25‐OHD level is a risk factor for acute invasive enterocolitis in children aged 1–3, and regular vitamin D supplementation and ample outdoor time demonstrated potential to guard against this ailment. Maintaining adequate serum 25‐OHD levels may help shorten illness and reduce antibiotic use.

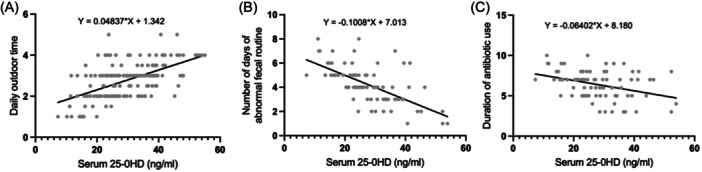

## 1 INTRODUCTION

Diarrhea is one of the leading causes of infectious fatalities among children below 5 years old worldwide, resulting in an estimated annual toll of 526,000 childhood deaths.^1^ The diarrhea mortality rate ranged between countries by over 685 deaths per 100,000 children.^2^ The World Health Organization has defined acute diarrhea as the occurrence of three or more episodes of loose or liquid stools per day, persisting for three or more days, though not exceeding 14 days.^3^ Based on its etiology, acute diarrhea is classified into infectious and noninfectious. Infectious diarrhea is attributed to infections within the intestinal tract due to viruses, bacteria, fungi or parasites, constituting the most prevalent cause of diarrhea,^4^, ^5^ with bacterial enteritis accounting for approximately 10%–15% of all diarrhea cases worldwide.^6^ Based on the pathogenic agents and mechanisms, infectious diarrhea can be further categorized into enterotoxigenic enteritis and invasive enteritis. Enterotoxigenic enteritis represents a form of secretory diarrhea driven by enterotoxin‐producing bacteria such as Vibrio cholerae and enterotoxin‐producing *Escherichia coli*, which proliferate solely within the intestinal lumen without infiltrating the intestinal mucosa. Comparatively, invasive enteritis arises from various invasive bacterial infections that induce exudative diarrhea, involving pathogens that directly invade the small intestine or colon walls, causing harm to the intestinal epithelium, triggering inflammation, epithelial necrosis and the formation of microabscesses, which then culminates in the development of ulcers and oozing lesions. Consequently, the discharged fecal matter exhibits characteristics resembling dysentery, containing substantial quantities of leukocytes and erythrocytes, accompanied by systemic manifestations of toxicity. The common pathogens implicated in invasive enteritis include *Shigella* spp., *Salmonella* spp., invasive *Escherichia coli*, *Campylobacter jejuni*, *Yersinia pestis*, and others.^7^, ^8^, ^9^ There are still considered as risk factors including no handwashing with soap, low rotavirus vaccine coverage, unsafe sanitation, unsafe water, zinc deficiency, childhood stunting, suboptimal breastfeeding, and vitamin A deficiency.^10^ Diarrhea may be accompanied by dehydration, electrolyte abnormalities and growth faltering.^6^

**Figure 1:**
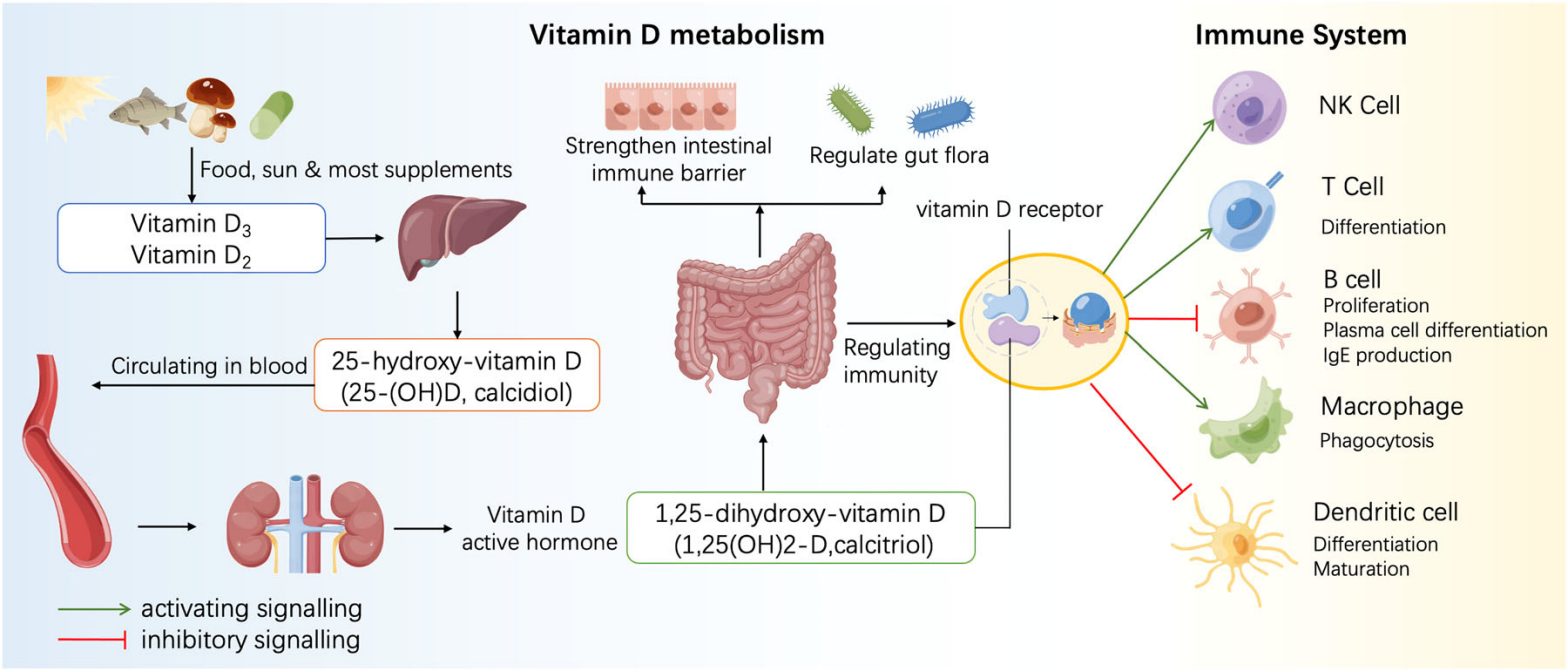
Metabolic processes and biological functions of vitamin D. Absorption of vitamin D in the body occurs primarily in the small intestine, followed by two hydroxylation reactions: the first in the liver and the second in the kidneys, as well as in many vitamin D‐responsive cells, including immune cells, to form the active form, calcitriol (1,25‐dihydroxycholecalciferol). Calcitriol acts on multiple tissues via the vitamin D receptor (VDR), modulating gut microbiome composition, barrier function, and mucosal immune responses.

**Figure 2:**
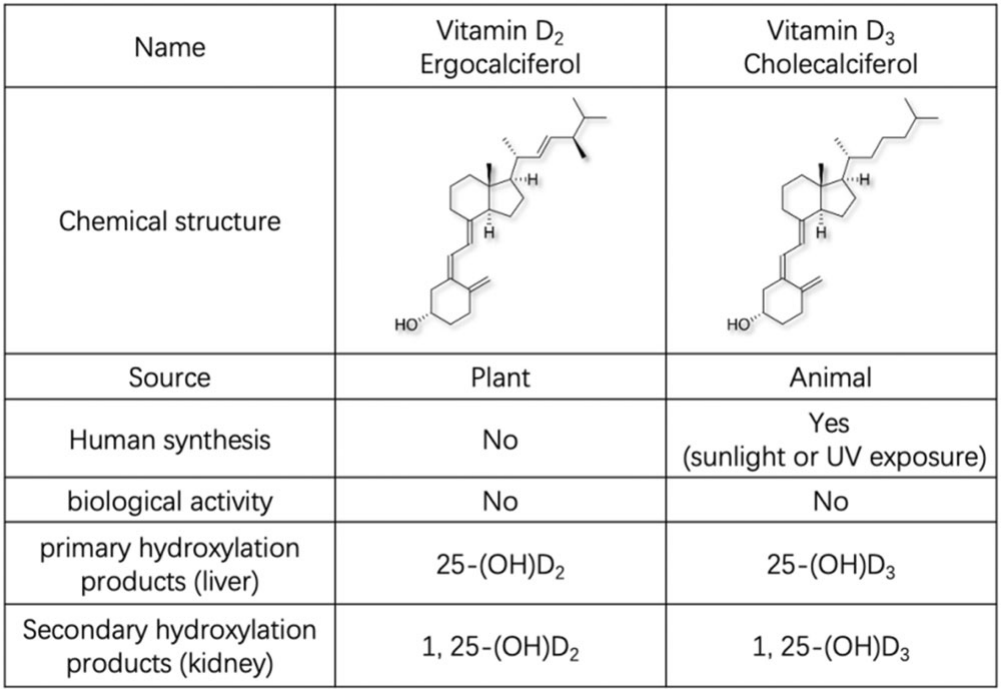
Classification and characterization of vitamin D. Vitamin D_2_ and vitamin D_3_ from different sources. Vitamin D_2_ is mainly found in plants and synthesized from ergosterol by UV irradiation. Vitamin D_3_ is primarily found in animals and synthesized from 7‐dehydrocholesterol through UV exposure. The body can also synthesize its own vitamin D_3_ through skin exposure to sunlight.

Vitamin D is a steroid hormone that is derived from its cholesterol precursor by the action of ultraviolet B radiation splitting a carbon‐to‐carbon bond. And ultraviolet B, which is largely blocked by the earth's ozone layer, is only received in countries away from the tropics by exposure to sunlight in summer months during the 3 to 4 h around midday.^11^, ^12^ Vitamin D plays a pivotal role as a major regulator of immune function and inflammation, exerting a modulatory influence on both adaptive and intrinsic immunity. This essential nutrient significantly impacts the development and functioning of critical immune cells, including T cells, dendritic cells, and regulatory T cells.^13^ Notably, vitamin D can stimulate the synthesis of anti‐inflammatory cytokines while concurrently restraining the expression of pro‐inflammatory cytokines.^14^ Its physiologically significant function extends to safeguarding the human host against assaults by bacterial and viral agents^15^ (Figure 1). Vitamin D_2_ and vitamin D_3_ are the two main forms of vitamin D (Figure 2). These two variants exhibit certain structural dissimilarities, with vitamin D_2_ having one additional methyl group and carbon‐carbon double bond compared to vitamin D3. They also differ in their origins: vitamin D_2_ is predominantly found in plant and fungal sources such as mushrooms and yeast, while vitamin D_3_ is mainly found in animal‐derived foods, including cod liver oil, egg yolks and dairy products. Existing literature has confirmed that vitamin D plays a role in regulating cell proliferation, differentiation, and apoptosis within the intestinal tract.^16^ Vitamin D deficiency precipitates a decline in the body's capacity to clear colonic bacteria, reduces the expression of tight junction proteins in intestinal epithelial cells, and enhances Th1 cell‐mediated intestinal inflammatory responses.^17^ In recent years, although studies investigating the association between vitamin D and human ailments have garnered considerable attention, only few studies have explored the connection between vitamin D levels and invasive enteritis in pediatric populations. In this present study, we measured and analyzed the serum vitamin D levels in young children aged 1–3 years with acute invasive enteritis in an attempt to identify strategies for the effective prevention and treatment of invasive enteritis in young children.

## 2 MATERIALS AND METHODS

### 2.1 Research object

This study is a prospective cohort study. We continuously recruited children aged 1–3 years diagnosed with acute invasive enteritis at the Pediatric Gastroenterology Department of Sichuan Provincial Maternal and Child Health Hospital from February 2021 to February 2022. The control group consisted of healthy toddlers aged 1–3 years who underwent routine health checks at our hospital during the same period, with no infection‐related risk factors in the past 2 weeks and no clinical or laboratory signs of infection. Basic information on vitamin D supplementation and outdoor activities in the 3 months before illness was collected via questionnaires, and clinical observations recorded the outcomes of the children with enteritis. Children who regularly supplemented with 600 IU of vitamin D daily (≥28 days/month) were classified into the regular supplementation group, while those who supplemented infrequently (≤10 days/month) were classified into the low‐frequency supplementation group.

Exclusion criteria included: those who supplemented vitamin D but did so insufficiently (daily intake <600 IU or monthly supplementation between 11 and 28 days); underlying conditions that could affect nutritional metabolism (such as birth gestational age ≤28 weeks, birth weight ≤1000 g, severe malnutrition, severe obesity, gastrointestinal developmental abnormalities, immunodeficiency, food protein allergy, inflammatory bowel disease, celiac disease, hyperthyroidism, diabetes, or history of intestinal surgery); antibiotic use within the 2 weeks before consultation; incomplete clinical data; or refusal of participation by the child or their family.

Anonymity was ensured for all participating children, and informed consent was obtained from their respective guardians.

### 2.2 Diagnostic criteria for acute invasive enteritis

In accordance with the Clinical Practice Guidelines for Infectious Diarrhea in Chinese Children,^8^ the diagnostic criteria for acute infectious diarrhea were fulfilled, which included the presence of mucopurulent pus‐blood stools accompanied by an increased stool frequency, the observation of a notable quantity of leukocytes and varying levels of erythrocytes during stool microscopy, a positive fecal culture result, or the co‐occurrence of fever, heightened blood leukocyte count and elevated C‐reactive protein (CRP) levels, while excluding alternative sources of infection.

### 2.3 Clinical data collection

A questionnaire encompassing various aspects such as gestational age at birth, birth weight, mode of delivery, birth season, urban or nonurban residence, maternal education, infant feeding practices, vitamin D supplementation and outdoor activities over the past 3 months was distributed to all participating children. For each child, monthly age, gender, weight and weight Z‐score were documented. Within the study group, ceftriaxone was administered at a dosage of 60 mg/kg per dose, once daily, accompanied by the collection of daily stool samples for routine and occult blood examinations, and parameters such as body temperature, the presence of vomiting, stool test results and the duration of antibiotic administration were also recorded.

### 2.4 Experimental methods

In the study group, fresh stool specimens were collected during initial visits for routine fecal (including stool white blood cell count, red blood cell count, pus cell count, fat droplets, parasites, etc.) and occult blood tests, along with blood samples for routine blood analyses (including white blood cell count, absolute neutrophil count, hemoglobin, red blood cell count, hematocrit, platelet count, etc.), C‐reactive protein (CRP), and 25‐OHD testing. The control group provided similar specimens during routine health care appointments. LIAISON XL automatic chemiluminescence immunoassay analyzer from Sorin (Italy) and LIAISON 25‐hydroxy total vitamin D assay kit from DiaSorin were used for the 25‐OHD test. All procedures were performed in strict accordance with the kits' manufacturer's instructions. According to the Practice Guidelines for Vitamin D Nutrition‐related Clinical Problems in Chinese Children, the vitamin nutritional status was graded according to the serum 25(0H)D levels, with serum 25‐OHD level of >20 ng/mL (50 nmol/L) regarded as normal, 12–20 ng/mL (30–50 nmol/L) as deficiency, ＜12 ng/mL (30 nmol/L) as severe deficiency.^18^

### 2.5 Statistical analysis

Statistical analysis was performed using the SPSS 25.0 software. Categorical data were presented as *n* (%), and group comparisons were made using the *χ* ^2^ test or Fisher's exact test. Continuous data were expressed as mean ± standard deviation (*x̅* ± *s*), and group comparisons were made using independent sample *t* tests for normally distributed data and independent sample Mann‐Whitney *U* tests for non‐normally distributed data. Additionally, Pearson correlation analysis was performed to explore bivariate correlations. Multifactorial analysis was accomplished through logistic regression analysis, and *p* < .05 was used to determine statistical significance.

## 3 RESULTS

### 3.1 General information

A total of 216 cases of acute diarrhea in young children aged 1–3 years, who were treated at the Pediatric Gastroenterology Department of Sichuan Maternal and Child Health Hospital from February 2021 to February 2022, were identified as resulting from invasive bacterial infections. Among these cases, 82 (38.0%) children with 49 (59.8%) males and 33 (40.2%) females met the inclusion criteria for the study group. The control group comprised 80 healthy toddlers, comprising 38 (47.5%) males and 42 (52.5%) females. As shown in Table 1, no statistically significant differences were observed between the two groups in terms of sex, age in months, weight, birth weight, gestational age at birth, mode of delivery, birth season, maternal education, or the proportion of urban residence (*p* > .05). However, distinct differences were evident in certain parameters. Compared to the control group, the study group had significantly lower proportion of infants who received exclusive breastfeeding during infancy (*p* < .05), and had reduced daily outdoor activity duration (*p* < .05).

**Table 1:** Comparison of clinical characteristics of children in the two groups [(*x̅±s*) or *n* (%)].

Characteristic	Study group (*n* = 82)	Control group (*n* = 80)	*t/Z/x* ^*2*^	*p*
Male	49 (59.8)	38 (47.5)	1.028	.08
Age, month	21.14 ± 5.77	21.24 ± 5.47	−1.869	.306
Weight, kg	11.64 ± 0.87	11.84 ± 0.90	−1.418	.158
Gestational age at birth	37.78 ± 2.24	37.71 ± 1.96	−0.649	.516
Birth weight	2.96 ± 0.61	2.97 ± 0.59	0.027	.978
Cesarean delivery	39 (47.6)	40 (50)	0.096	.756
Winter‐borns	20 (24.4)	20 (25)	2.206	.531
College‐educated mothers	6 (7.3)	5 (6.3)	0.489	.937
Urban resident	60 (73.2)	57 (71.3)	0.074	.785
Infant feeding methods				
Exclusive breastfeeding	22 (26.8)	46 (57.5)	16.755	<.001
Artificial	18 (22.0)	14 (17.5)		
Mix	42 (51.2)	20（25.0)		
Outdoor activity time, hours/day	2.59 ± 0.92	3.04 ± 0.70	−3.603	<.001
Clinical Presentation				
Fever	34 (41.5)	‐	‐	‐
Emesis	25 (30.5)	‐	‐	‐
Hematological indicators				
White blood cell count (10^9^/L)	16.27 ± 2.52	7.24 ± 1.27	28.894	<.001
Neutrophil rate (%)	68.46 ± 5.74	34.47 ± 5.17	36.061	<.001
Hemoglobin (g/L)	116.02 ± 11.09	117.33 ± 9.90	−0.787	.433
Platelets (10^9^/L)	252.94 ± 9.32	244.73 ± 9.17	0.628	.531
CRP (mg/dL)	37.69 ± 12.35	1.73 ± 1.04	26.267	<.001

Within the study group, 24 (29.3%) children were hospitalized, while 58 (70.7%) received outpatient care. Fever was present in 34 (41.5%) cases, and vomiting was observed in 25 (30.5%) cases. Key hematological indicators, such as leukocyte count, neutrophil ratio, and CRP level, were substantially elevated in the study group compared to the control group (*p* < .05). However, no statistically significant distinction was identified in peripheral hemoglobin concentration and platelet count between the two groups (*p* > .05). All children within the study group were empirically treated with ceftriaxone, administered at 60 mg/kg per dose once daily. Among the 82 children, 70 (85.4%) cases underwent fecal culture, resulting in positive findings for 59 (84.3%) cases. Etiological analysis all indicated Gram‐negative bacteria infection (59/59, 100%), including *Salmonella* (30/59, 50.8%), *Escherichia coli* (12/59, 20.3%), *Shigella* (6/59, 10.2%), *Campylobacter jejuni* (5/59, 8.5%), *Yersinia* (4/59, 6.8%), and *Klebsiella pneumoniae* (2/59, 3.4%). In contrast, 11 cases yielded negative culture results, while 12 cases lacked fecal culture data. Notably, these 23 cases exhibited common clinical characteristics, including mucopurulent stools, high fever (temperature ≥ 39°C), elevated blood leukocyte counts, and CRP levels, with exclusion of other potential sources of infection, thereby meeting clinical diagnostic criteria. Subsequent to treatment, all children experienced successful recovery.

### 3.2 Comparison of vitamin D supplementation status and serum 25‐OHD levels and distribution between two groups of young children

Serum 25‐OHD levels exhibited a statistically significant decrease in the study group compared to the control group (*p* < .01). Similarly, the proportion of individuals undergoing regular vitamin D supplementation was markedly lower within the study group than in the control group (*p* < .05). Importantly, no severe vitamin D deficiency (25‐OHD ≤ 5 ng/mL) was observed within either the study group or the control group, and no statistically significant differences were detected in the distribution of normal, insufficient and deficient statuses of serum 25‐OHD levels among the young children within the two groups (*p* > .05) (Table 2).

**Table 2:** Comparison of vitamin D supplementation, serum 25‐OHD levels and distribution between the two groups of children [(*x̅±s*) or *n* (%)].

Variables	Study Group (*n* = 82)	Control group (*n* = 80)	*t/x* ^*2*^	*p*
Regular vitamin D supplementation (%)	48 (58.5)	62 (77.5)	6.681	.010
Serum 25‐OHD (ng/mL)	27.95 ± 9.91	32.76 ± 10.23	3.040	.003
Serum 25‐OHD level distribution				
Normal (>20 ng/mL)	66 (80.5)	70 (87.5)	1.542	.477
Insufficient (12–20 ng/mL)	12 (14.6)	8 (10)		
Deficiency (<12 ng/mL)	4 (4.9)	2 (2.5)		

### 3.3 Comparison of serum 25‐OHD levels between the regular vitamin D supplementation group and the low‐frequency supplementation group

Over the course of 3 months, a daily dose of 600IU of vitamin D was administered. Participants were categorized into regular supplementation and low‐frequency supplementation groups based on their monthly supplementation time, with regular supplement defined as ≥28 days and low‐frequency supplement defined as ≤10 days. Out of the 82 children in the study group, 48 were classified as regularly supplemented and 34 as infrequently supplemented. In the control group of 80 children, 62 were regularly supplemented and 18 were infrequently supplemented. Comparative analysis showed that the serum 25‐OHD level in regular supplementation group (*n* = 110) was significantly higher than that in infrequently supplementation group (*n* = 52) (*p* < .05) (Table 3).

**Table 3:** Comparison of serum 25‐OHD levels between the regular supplementation group and the low‐frequency.

Variable	Low‐frequency supplementation group (*n* = 52)	Regular supplementation group (*n* = 110)	*t*	*p*
Serum 25‐OHD (ng/mL)	26.58 ± 8.48	32.09 ± 10.67	3.542	<.001

### 3.4 Correlation analysis of serum 25‐OHD level with outdoor activity time and duration of abnormal fecal routine and antibiotic use in the study group

**Figure 3:**
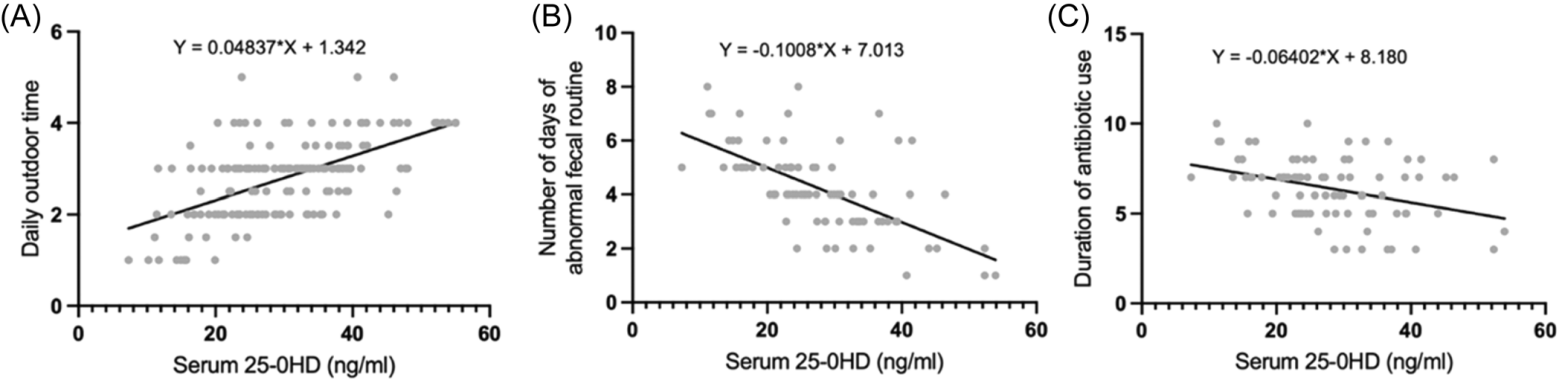
(A) Correlation analysis between outdoor activity time and serum 25‐OHD level (*r* = .588, *p* < .001). (B) Analysis of the correlation between serum 25‐OHD level and the duration of abnormal stool routine (*r* = −.683, *p* < .001). (C) Analysis of the correlation between serum 25‐OHD levels and the duration of antibiotic use (*r* = −.366, *p* < .001). ‘The duration of abnormal stool routine’ refers to the length of time that children who continue to exhibit diarrhea symptoms or show elevated white blood cell counts in routine stool tests.

Among the 162 toddlers, the average 25‐OHD level was 30.33 ± 10.32 ng/mL, their daily outdoor activity averaged 2.81 ± 0.85 h/day, and a significant positive correlation between these two factors was observed (*p* < .05). Within the study group of 82 toddlers, the average 25‐OHD level was 27.95 ± 9.91 ng/mL, and the mean duration of abnormal stool routines and antibiotic use were 4.20 ± 1.57 days and 6.39 ± 1.73 days, respectively. Furthermore, a significant negative correlation was found between serum 25‐OHD levels and both the duration of abnormal stool routines and antibiotic use (*p* < .05) (Figure 3).

### 3.5 Analysis of risk factors for acute invasive enteritis

Baseline variables that were considered clinically relevant or were significantly associated with the outcome of enteritis in the univariate analysis were tentatively included in the multivariate model. Considering the available number of case events, the sample size evaluation criteria (the principle of Events per variable (EVP) = 10% in sample size estimation),^19^ and ensuring the simplicity of the model, 6 variables were ultimately selected, comprising gender, age, birth season, infant feeding methods, outdoor activity time and regular vitamin D supplementation included in our logistic regression model, as illustrated in Table 4.

**Table 4:** Multifactorial regression analysis for invasive enteritis.

Variables	*β*	OR	95% CI	*p*
Gender (male)	.472	1.604	0.791–3.251	.19
Age, month	.051	1.052	0.986–1.123	.126
Winter‐borns	−.196	0.822	0.353–1.913	.649
Exclusive breastfeeding	−1.354	0.258	0.125–0.532	<.001
Outdoor activity time (h/day)	−.77	0.463	0.297–0.722	<.001
Regular vitamin D supplementation	−1.033	0.356	0.162–0.781	.01
Constant	2.166	8.725	‐	.022

The results of the multivariate logistic regression analysis indicated that infant feeding methods, outdoor activity time, and regular vitamin D supplementation were identified as independent factors influencing the occurrence of acute invasive enteritis. Exclusive breastfeeding during infancy was associated with a 0.742 decrease in the risk of developing the disease (*p* < .05). Additionally, each additional hour of daily outdoor activity was linked to a 0.537 decrease in the risk of disease development (*p* < .05). Furthermore, individuals who regularly supplemented with vitamin D had a 0.644 lower risk of developing the disease compared to those who did not regularly supplement with vitamin D (*p* < .05).

## 4 DISCUSSION

Gastroenteritis is the most common cause of acute diarrhea. It arises from infections caused by bacteria, viruses, and parasites, exhibiting clinical manifestations such as vomiting, nausea, fever, and abdominal pain. The incidence of this condition varies based on age, social, geographic factors, and environmental hygiene standards. The underlying etiology can be inferred through a comprehensive analysis of epidemiological factors, clinical attributes, physical examination outcomes, and the pathophysiological mechanisms of the implicated pathogen.^8^, ^20^ Notably, among bacterial contributors, *Campylobacter*, *Shigella*, *Salmonella* and pathogenic *Escherichia coli* are the primary agents responsible for acute invasive gastrointestinal infections.^21^ The main mechanism by which vitamin D is involved in intrinsic immunity is by influencing the expression of cytokines, antimicrobial proteins and pattern recognition receptors.^14^ There is conclusive evidence showing that vitamin D supplementation can reduce the incidence of infectious diseases in the pediatric population.^22^ For instance, a correlation between maternal and neonatal vitamin D deficiency and sepsis in term neonates has been reported.^23^ In addition, recent studies have further indicated the potential of vitamin D supplementation in mitigating COVID‐19 infections and their severity, particularly among the elderly and individuals exhibiting low serum 25‐OHD levels.^24^

Vitamin D plays a pivotal role in maintaining intestinal equilibrium within the gastrointestinal tract by orchestrating microbial composition, bolstering barrier function, and modulating mucosal immune responses.^25^, ^26^, ^27^ The integrity of the intestinal epithelial barrier holds paramount significance in the host's resilience against pathogens.^28^ Active vitamin D contributes to the expression of tight junction proteins within the intestinal epithelium, thereby safeguarding the integrity of the mucosal barrier. Notably, a deficiency in vitamin D fosters a pro‐inflammatory state, heightening susceptibility to intestinal inflammation.^14^ A prior investigation conducted by our study group revealed a potential correlation between serum 25‐OHD levels in mothers and preterm infants and the incidence of necrotizing small intestinal colitis in the latter.^29^ Furthermore, Bucak et al. highlighted that reduced serum vitamin D levels could be a potential risk factor for rotavirus‐induced diarrhea in children,^27^ Gao et al. reported a correlation between vitamin D deficiency and Helicobacter pylori infections in children aged 6 to 36 months,^28^ and the study by Lobo et al. highlighted the potential of vitamin D to counteract the intestinal epithelial barrier malfunction caused by *Campylobacter jejuni* infection, suggesting its promise as a therapeutic avenue for managing *Campylobacter jejuni* infection in both humans and animals.^24^ Our present study identified a significant difference in serum 25‐OHD levels between children aged 1‐3 years afflicted with acute invasive enteritis and those within the control group. Notably, these lower levels exhibited a strong and inverse correlation with both the duration of atypical stool patterns and the length of antibiotic administration following effective treatment. This observation implies a potential connection between diminished serum 25‐OHD levels and the onset of this ailment. Furthermore, it suggests that maintaining optimal serum 25‐OHD levels could potentially yield benefits in terms of reducing the disease's duration and the necessary duration of antibiotic use.

**Figure 4:**
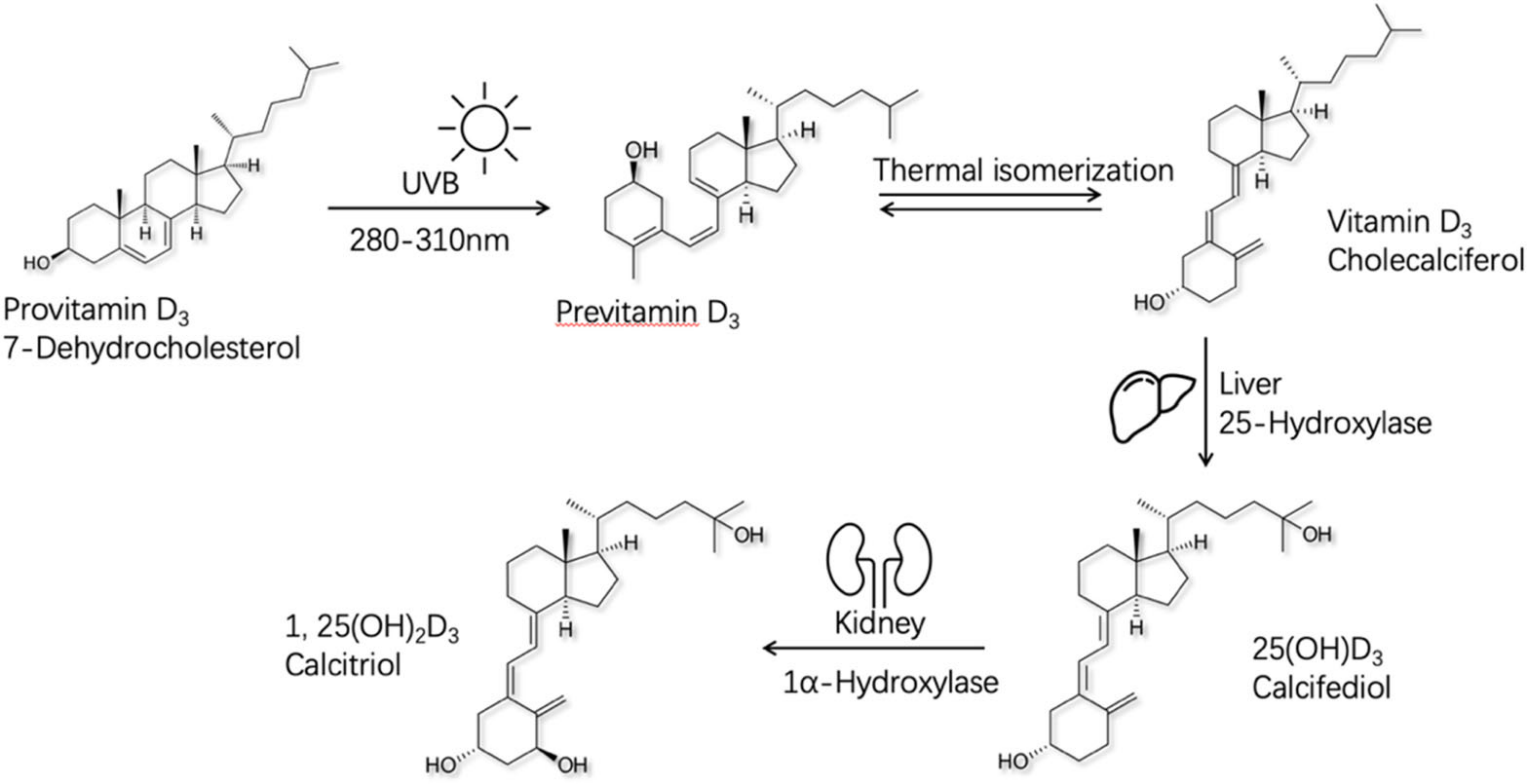
Vitamin D_3_ synthesis occurs in the skin upon exposure to UVB radiation, a form of electromagnetic radiation in the 280‐310 nanometer wavelength range emitted by the sun. This radiation initiates the opening of the B‐loop of 7‐dehydrocholesterol, a precursor to cholesterol, leading to the formation of pro‐vitamin D_3_. Subsequent heat‐induced isomerization transforms pro‐vitamin D_3_ into vitamin D_3_. The variants of both vitamin D_2_ and D_3_ undergo conversion into 25‐hydroxyvitamin D (25(OH)D), the primary gauge of the body's vitamin D levels, via the enzymatic action of 25‐hydroxylase in the liver. The ensuing step involves the transformation of 25(OH)D through the enzymatic action of 1‐hydroxyvitamin D into 1,25‐dihydroxy vitamin D (1,25(OH)_2_D)—also known as calcitriol—the most potent form of vitamin D and is facilitated by the enzyme 1alpha‐hydroxylase located in the kidneys.

Synthesis of vitamin D_3_ through skin exposure to ultraviolet B (UVB) light is one of the major ways the body acquires vitamin D, which accounts for 90% of vitamin D sources.^30^ Vitamin D_3_ undergoes two hydroxylation reactions in the liver and kidneys to transform into its active form of vitamin D (Figure 4). In this study, we found a significant positive correlation between outdoor activity time and serum 25‐OHD levels. Additionally, each additional hour of daily outdoor activity was linked to a 0.537 decrease in the risk of acute invasive enteritis development (*p* < .05). This suggests that getting enough sunlight for vitamin D synthesis may help prevent this condition. Natural dietary sources of vitamin D are scarce, with the UK Scientific Advisory Committee on Nutrition (SACN) acknowledging the challenge of meeting recommended vitamin D intake solely through food and suggesting population‐wide strategies to address this issue.^31^ In this present study, we found that serum 25‐OHD levels were significantly higher in the regular vitamin D supplementation group compared to the low‐frequency supplementation group. Children aged 1–3 years who regularly supplemented with vitamin D had a 0.644 lower risk of developing the disease compared to those who did not regularly supplement with vitamin D (*p* < .05). This finding emphasizes the potential of routine vitamin D supplementation and elevated serum 25‐OHD levels in mitigating the risk of acute invasive enteritis among children aged 1–3 years. Certain microorganisms within breast milk may influence and shape the infant gut microbiota, playing a pivotal role in regulating gut barrier integrity and fostering immune system development.^32^ Various bioactive factors in human milk contribute to health status during and beyond infancy.^33^ Our study revealed that exclusive breastfeeding during infancy was associated with a 0.742 decrease in the risk of developing the disease (*p* < .05), suggesting that exclusive breastfeeding during infancy might confer protective effects on intestinal immunity beyond infancy.

Vitamin D deficiency has emerged as a significant public health concern. A European study involving 55,844 individuals unveiled that 40.4% exhibited serum 25‐OHD levels below 20 ng/mL (50 nmol/L), with 13.0% displaying levels below 12 ng/mL (30 nmol/L).^31^ A survey by Herrick et al. revealed that vitamin D deficiency and insufficiency affected 5.0% and 18.3% of the US population aged ≥1 year from 2011 to 2014, respectively.^34^ Young children are particularly susceptible to acute invasive enteritis and vitamin D deficiency among the vulnerable groups. Our study highlights the pivotal role of regular vitamin D supplementation as an independent protective factor for acute invasive enteritis in children aged 1–3 years. Serum vitamin D levels are influenced by many factors, prompting the recommendation of tailored vitamin D supplementation regimens based on individual characteristics such as race, gender, age, dietary habits, season, and local sunlight exposure. Advising consistent vitamin D supplementation for all young children, coupled with appropriate outdoor activity and periodic monitoring of serum 25‐OHD levels to maintain them within a specified range, should be encouraged. Beyond its potential to reduce the risk of acute invasive enteritis, maintaining adequate serum vitamin D levels could potentially contribute to shortening the duration of illness and the need for antibiotic treatment when the ailment does occur.

## 5 CONCLUSION

Low serum 25‐OHD levels were identified as a risk factor associated with acute invasive enteritis in children 1–3 years old, and regular vitamin D supplementation and ample outdoor time demonstrated potential to guard against this ailment in the same age group. Further research is needed to ascertain the specific role and mechanism of sufficient serum vitamin D levels in preventing acute invasive enteritis.

## AUTHOR CONTRIBUTIONS

**Lingrong Yang**: Conceptualization; data curation; methodology; writing—original draft. **Yu Fang**: Data curation; visualization; writing—original draft. **Jinyu Zheng**: Data curation; methodology. **Qiaoying Zhu**: Data curation; methodology. **Li Tang**: Data curation; methodology. **Fu Xiong**: Conceptualization; funding acquisition; project administration; writing—review and editing.

## CONFLICT OF INTEREST STATEMENT

The authors declare that the research was conducted in the absence of any commercial or financial relationships that could be construed as a potential conflict of interest.

## ETHICS STATEMENT

The study was approved by the Ethics Review Committee of Sichuan Maternal and Child Health Hospital (20230331‐030) and was conducted following the ethical procedures outlined in the Declaration of Helsinki.

## PATIENT CONSENT STATEMENT

Anonymity was ensured for all participating children, and informed consent was obtained from their respective guardians.

## Data Availability

All raw data and code are available upon request.
